# Efficacy of *Cuminum cyminum* essential oil on *FUM1* gene expression of fumonisin-producing *Fusarium verticillioides* strains

**Published:** 2015

**Authors:** Ali Reza Khosravi, Hojjatollah Shokri, Ali Reza Mokhtari

**Affiliations:** 1 *Mycology Research Center, Faculty of Veterinary Medicine, University of Tehran, Tehran, I. R. Iran*; 2 *Faculty of Veterinary Medicine, Amol University of Special Modern Technologies, Amol, I. R. Iran*

**Keywords:** *Fusariumverticillioides*, *Fumonisin*, *Cuminumcyminum*, *RT*-*PCR*, *FUM1gene*

## Abstract

**Objectives:** The purpose of this study was to evaluate the effect of *Cuminum cyminum* (*C. cyminum*) essential oil on the growth and *FUM1 *gene expression of fumonisin-producing *Fusarium verticillioides* (*F. verticillioides*) strains isolated from maize.

**Materials and Methods:** All fungal strains were cultured on potato dextrose agar (PDA) slopes at 30°C for 7 days. The antifungal activity was evaluated by broth microdilution assay. One set of primers was *F. verticillioides* species specific, which selectively amplified the intergenic space region of rDNA. The other set of primers was specific to* FUM1* gene region of fumonisin-producing *F. verticillioides. FUM1 *transcript levels were quantified using a reverse transcription-polymerase chain reaction (RT-PCR) protocol.

**Results:** Minimum inhibitory concentration (MIC) values of *C. cyminum* oil against *F. verticillioides* strains varied from 0.195 to 0.781 µl.ml^-1^ (mean MIC value: 0.461 µl.ml^-1^) indicating 54.5% of the fungal strains inhibited at 0.390 µl.ml^-1^. PCR analysis of *FUM1* gene expression revealed that DNA fragment of 183 bp was amplified in all the isolates of *F. verticillioides* before treatment with *C. cyminum* essential oil. Based on RT-PCR analyses, reduction in the expression of fumonisin biosynthetic genes was significant only for *FUM1 *gene (*p*<0.05), while no effect was observed on *ITS* gene.

**Conclusions: **This study showed that all *F. verticillioides* isolates were susceptible to *C. cyminum* essential oil, indicating a significant reduction in the growth of fungal isolates. In addition, this oil completely inhibited the expression of *FUM1 *gene in concentrations dose-dependently.

## Introduction

The genus *Fusarium* represents one of the major fungal genera, which are ubiquitous in distribution and are found frequently on freshly harvested and stored agricultural commodities such as cereals. *Fusarium verticillioides* (*Gibberella moniliformis*, *Gibberella fujikuroi* mating population A) is the main source of fumonisins, a group of toxins which contaminates commodities, causing chronic and acute diseases in humans (Gonzalez-Jaen et al., 2004 ▶). In addition, fumonisins cause several diseases in animals such as leukoencephalomalacia in horses, pulmonary edema in swine and hepatic cancer in rats. They are considered to be Group 2B carcinogens by the International Agency for Research on Cancer (Nelson et al., 1993 ▶).

The importance of *F. verticillioides* as a causal agent of several diseases in cereals, and the production of fumonisins during the course of host infection, even when no visible symptoms are observed, emphasize the need to control both pathogen growth and the production of fumonisin. In order to develop efficient strategies to achieve these goals, it is crucial to have more information regarding the population structure and variability of the species as well as the biosynthetic and regulatory pathways of mycotoxin production. This information could also be used to develop rapid, sensitive, and specific assays to detect the presence of mycotoxigenic species or lineages in the early stages of infection and to prevent mycotoxins entering the food chain.

Various PCR assays have been developed for the identification of toxigenic species of *Fusarium*. Some of them are based on single copy genes directly involved in mycotoxin biosynthesis while others are species-specific (Gonzalez-Jaen et al., 2004 ▶; Mule et al., 2004 ▶). Biochemical analyses indicated that fumonisins are products of a polyketide synthase (PKS) gene called *FUM1* (Desjardins et al., 2002 ▶; Proctor et al., 2003 ▶) whose regulation occurs at the transcriptional level (Seo et al., 2001 ▶). Recent reports indicate significant correlation between *FUM1 *transcripts, quantified by reverse transcription-PCR (RT-PCR), and phenotypic fumonisin production (Jurado 2006 ▶; Lopez-Errasquın et al., 2007 ▶). The quantitation of *FUM1 *transcripts by RT-PCR permits a sensitive and specific approach to evaluate the effects of various treatments on fumonisin biosynthesis.

Numerous studies have been performed on the plant compounds with the intent to reduce the growth of mycotoxin-producing fungi, inhibit toxin production, and suppress the major toxin encoded genes in these organisms. *Cuminum cyminum* (Apiaceae) is an annual herbaceous plant (height: 15-50 cm) with green seeds which have aromatic characteristics. It is believed to the natives of the Mediterranean and Near Eastern regions. It is applied in Iranian folk medicine since more than 200 years ago. It has been shown that its fruits have medicinal application in treatment of diarrhea, toothache, and epilepsy (Zargari, 1994 ▶). It also has diuretic, carminative, emmanogogic, and antispasmodic properties (Janahmadi et al., 2006 ▶). Besides its use in traditional medicine in the treatment of some ailments, *C. cyminum* is widely used in food. It is one of the popular spices regularly used as a flavoring agent and possesses numerous antimicrobial activities (Iacobellis et al., 2005 ▶; Nostro et al., 2005 ▶). Major constituents in *C. cyminum* essential oil are gamma-terpinene, 2-methyl-3-phenyl-propanal, myrtenal, and glucopyranosides (Jalali-Heravi et al., 2007 ▶). There is no report regarding the effect of *C. cyminum* essential oil on fumonisin-producing *F. verticillioides* strains. The objective of this study was to evaluate the effect of *C. cyminum* essential oil on the growth and *FUM1 *gene expression in fumonisin-producing strains of* F. verticillioides*. These relationships are important as they clarify the ecophysiological basis for the establishment and survival in crop residue and for subsequent spread and infection of maize.

## Materials and Methods

**Fungal isolates**

Fourteen *F. verticillioides* strains, F1-14, were prepared from Mycology Research Center, University of Tehran, Tehran, Iran. All fungal strains, originally isolated from the maize farm in Iran, were cultured on potato dextrose agar (PDA,* Merck Co., Darmstdt, Germany*) slopes at 30°C for 7 days. 

Slopes were flooded with phosphate-buffered saline (PBS) containing 0.05% Tween 80. Fungal growth was gently probed and the resulting suspension was removed and mixed thoroughly by a vortex mixer. After the settling of the large rparticles, suspension was adjusted by hemocytometer to final inoculums concentration of 0.5×10^6^ cell.ml^-1^.

**Extraction of C.** ***cyminum*** **oil**

The seeds of *C. cyminum* were dried at room temperature (20-23ºC) and ground into a powder using a blender. The essential oil was obtained from 50 g of powdered seeds by water-distillation using a Clevenger-type system for 3 h. The aqueous phase was extracted three times with 50 ml dichloromethane. The organic phase was dried with sodium sulphate and filtered. The solvent was allowed to evaporate until dryness. Oil sample was stored at −25ºC in a sealed glass vial (FarmacopeiaBrasileira, 2000 ▶).

**Antifungal activity assay**

Broth microdilution testing was performed based on M38-A protocol for filamentous fungi (NCCLS, 2002). Briefly, a series of doubling dilutions of cumin oil ranging from 0.0975 to 3.12 µl.ml^-1^ was prepared in a 96-well microdilution plate, with a final concentration of 0.001% (v/v) Tween 80 to enhance cumin oil solubility. After the addition of inocula (prepared as described above), plates were incubated at 35°C for 48 h. Minimum inhibitory concentrations (MICs) were determined visually with the aid of a reading mirror, according to NCCLS guidelines. Minimum fungicidal concentrations (MFCs) were also determined by using 0.01 ml from each well without visible growth onto PDA plates. MFCs were determined as the lowest concentration resulting in no growth on subculture. Each experiment was repeated in duplicate.

**Treatment of** ***F. verticillioides*** **strains with** ***C. cyminum*** **oil**

A 10 μl spore suspension was prepared from 7-day-old PDA cultures, of each *F. verticillioides* strain was inoculated into 250 μl of fumonisin-inducing liquid medium exposed to different concentrations of *C. cyminum* essential oil (0.195 and 0.390 µl.ml^-1^) and incubated at 20°C for 7 days. Mycelia were harvested by filtration through Whatman No.1 paper (*Whatman International, Ltd, England*), frozen in liquid nitrogen and kept at -70°C for RNA isolation. The fungal filtrates were used to analyze fumonisin contents by high-performance liquid chromatography (HPLC) according to the manufacturer’s protocol (Lopez-Errasquin et al., 2007 ▶).

**DNA extraction and polymerase chain reaction (PCR) amplification**

Genomic DNA of the fungal strains was obtained using the “GeneJET™ Plant Genomic DNA Purification Mini Kit” (Fermentas) following the manufacturer's instructions. DNA samples from *F. verticillioides* isolates as well as from the control sample (*Aspergillus flavus*) were subjected to PCR analyses using *Fusarium* species specific *ITS* and *FUM1* primers: *ITS* Forward: 5'-AACTCCCAAACCCCTGTGAACATA-3', *ITS* Reverse: 5'-TTTAACGGCGTGGCCGC-3'), and *FUM1* Forward: 5´-GCGGGAATTCAAAAG-3', *FUM1* Reverse: 5´-GAGGGCGCGAAACGGATCGG-3') (Sreenivasa et al., 2006). The PCR reaction was carried out in 25 μl volume containing 10 ng of DNA sample, 10X Taq polymerase buffer (*AB-gene Housse, UK*), 25 mM MgCl2, 2 mMdNTPs, 20 pmol of each forward and reverse primer, and 0.5 μl (3U.μl^-1^) of Red Hot Taq. The PCR reactions were performed using Thermocycler (*Techne, TC-512, UK*). Samples were heated at 94ºC for 3 min and then subjected to 32 cycles of 1 min at 95ºC (denaturation), 1 min at 60ºC (annealing) and 3 min at 72ºC (extension). The final extension was set at 72°C for 5 min. Ten μl of the PCR product was electrophoresed on 1.5% agarose gel, stained with ethidium bromide, illuminated, and documented using Biorad UV Transilluminator. 

**RNA isolation and reverse transcription (RT)-PCR**

Fungal total RNA was isolated using the “GeneJET Plant RNA PurificationMini Kit” (Fermentas) according to the manufacturer's instructions and stored at −80°C. DNAse I treatment to remove genomic DNA contamination from the samples was performed using RibolockRNase Inhibitor following the manufacturer's instructions. First strand cDNA was synthesized using the “RevertAid First Strand cDNA Synthesis Kit” (Fermentas). Each 20 μl reaction contained 500 ng of total RNA, 1 μl of oligo d(T)16 (50 μM), 4 μl of 5×RT-PCR buffer, 1 μl of MgCl2 (25 mM), 2 μl of dNTPs (10 mM), 2 μl of DTT (100 mM), 1 μl of RNAse inhibitor (20 U.μl^-1^), 1 μl of RevertAid M-MulV Reverse Transcriptase (200 U.μl^-1^), and sterile DEPC water up to the final volume. Synthesis of cDNA was performed in a Thermocycler (*Techne, TC-512, UK*) according to the following procedure: 94ºC for 4 min and then subjected to 35 cycles of 1 min at 94ºC (denaturation), 1 min at 58ºC (annealing) and 1 min at 72ºC (extension). The final extension was set at 72°C for 10 min. The cDNA samples were kept at −20°C. Samples incubated in the absence of reverse transcriptase were used as controls (Jurado et al., 2010 ▶; Marín et al., 2010 ▶).

**Statistical analysis**

The data of fungal growth and gene expression were subjected to the analysis of variance (One-way ANOVA) in Tukey range. The differences with *p*<0.05 were considered significant.

## Results

 Our findings showed that all isolates were susceptible to the essential oil, indicating a significant reduction in the growth of fungal isolates. The MIC values of *C. cyminum* oil against *F. verticillioides* strains varied from 0.195 to 0.781 µl.ml^-1^ (mean MIC value: 0.461 µl.ml^-1^) representing 54.5% of the strains inhibited at 0.390 µl.ml^-1^ (Table 1). *C. cyminum* oil exhibited a remarkable fungicidal activity against the fungal isolates with MFC values ranging from 0.390 to 1.56 µl.ml^-1^. 

**Figure 1:**
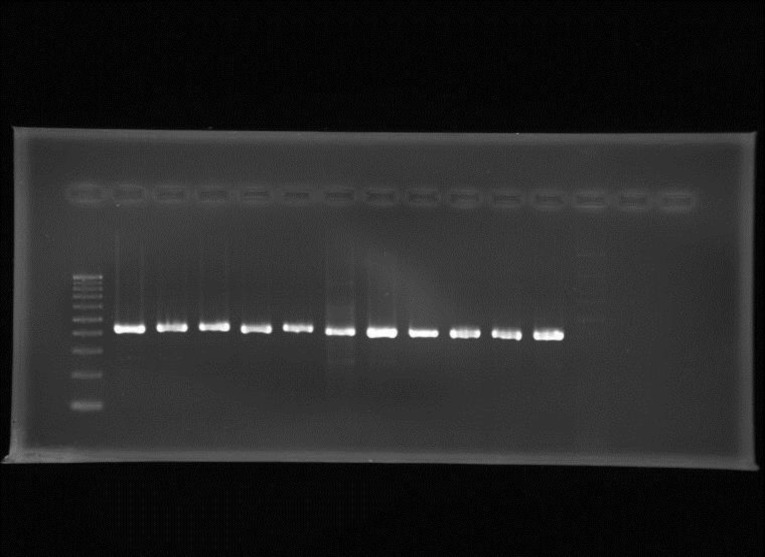
Agarose gel showing amplified products using primers for *ITS* region (431 bp). Lane marker (left): 100bp DNA ladder. Lanes 1-11 (from left to right): *Fusarium verticillioides* (F1-11)*. *Lane 12: *Aspergillus flavus*

**Figure 2:**
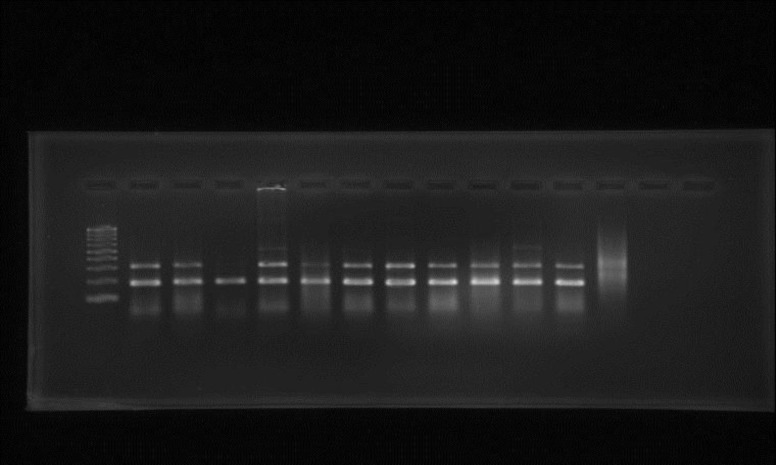
Agarose gel showing amplified products using primers for *FUM1* region (183 bp) before treatment with *C*. *cyminum* essential oil. Lane marker (left): 100bp DNA ladder. Lanes 1-11 (from left to right): *Fusarium verticillioides* (F1-11)*. *Lane 12: *Aspergillus flavus*

 In PCR analysis, all *F. verticillioides* strains were positive for the *ITS* region and exhibited the expected 431 bp DNA fragment except *A. flavus*, which was used as control (Figure 1). In addition, all the tested strains of *F. verticillioides* revealed the *FUM1* gene expression and amplified fragment with the molecular size of 183 bp, corresponding to the expected molecular size of the *FUM1* gene sequence, while this band could not be detected in the control sample (*A. flavus*) (Figure 2). 

**Figure 3:**
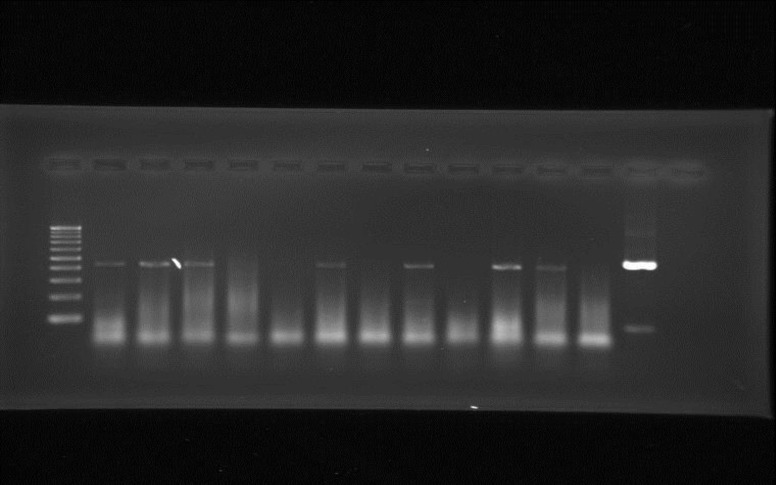
Agarose gel showing amplified products using primers for *ITS* and *FUM1* regions after treatment with *C*. *cyminum* essential oil. Lane marker (left): 100bp DNA ladder. Lanes 1-11 (from left to right): *Fusarium verticillioides* (F1-11)*. *Lane 12: *Aspergillus flavus*

 In order to evaluate the effect of *C. cyminum* oil on expression of *ITS* and *FUM1 *genes, *F. verticillioides* isolates were cultured on fumonisin-inducing liquid medium in presence of *C. cyminum* oil (0.195 and 0.390 µl.ml^-1^) at 20°C for 7 days. The results revealed that *C. cyminum* oil completely inhibited the expression of *FUM1 *gene in both concentrations dose-dependently (Figure 3). Based on the statistical analyses, reduction in the expression of fumonisin biosynthetic genes was significant only for *FUM1 *gene (*p*<0.05), while no effect was observed on *ITS* gene.

## Discussion

The antimicrobial properties of volatile aromatic oils from plants have been recognized since antiquity. Here, we evaluated the antifungal activity of essential oil obtained from *C. cyminum* against fumonisin-producing strains of* F. verticillioides*. Our results confirmed that all of the tested isolates were susceptible to the essential oil, indicating a significant reduction in the growth of fungal isolates. The rate of growth reduction was directly proportional to the concentrations of tested oil in the medium. In fact, higher concentrations of the oil led to lower fungal developments. As illustrated in Table 1, MIC values of *C. cyminum* oil against *F. verticillioides* strains varied from 0.195 to 0.781 µl.ml^-1^ (mean MIC value: 0.461 µl.ml^-1^) representing 54.5% of the strains inhibited at 0.390 µl.ml^-1^.

In addition, *C. cyminum* oil exhibited a remarkable fungicidal activity against the fungal isolates with MFC values ranging from 0.390 to 1.56 µl.ml^-1^. Previous studies showed that *C. cyminum* is effective on the growth of some microorganisms including fungi (Megalla et al., 1980 ▶; Naeini et al., 2009 ▶; Mohammadpour et al., 2012 ▶). In a study by Naeini et al. (2010), ▶ high *anti*-*Fusarium* activity was found at a very low concentration of *C. cyminum* oil against non-toxigenic (*F. solani*, and *F. oxysporum*) and toxigenic (*F*. *verticillioides*, *F. poae*, and *F. equiseti*) species. In agreement with our findings, Salehi Surmaghi (2006) ▶ reported strong antifungal activity of *C. cyminum* against *Fusarium* species.

**Table 1:** Antifungal susceptibility of *Cuminum cyminum* essential oil against *Fusarium verticillioides* isolates (µl.ml^-1^).

**Strains**	**F1 **	F2	F3	F4	F5	F6	F7	F8	F9	F10	F11	F12	F13	F14
**Value**														
MIC	0.195	0.781	0.781	0.390	0.390	0.390	0.781	0.390	0.195	0.390	0.390	0.195	0.781	0.390
MFC	0.390	1.56	1.56	0.781	0.781	0.781	1.56	0.781	0.390	0.781	0.781	0.390	1.56	0.781

Early and specific detection of toxigenic fungi lead to the better understanding of the pathogens, which pave the way of effective disease control. The PCR assay provides a useful tool for rapid and sensitive detection and differentiation of potential mycotoxin-producing fungi. In this study* F. verticillioides *isolates were subjected to PCR analysis using *ITS* genus specific and *FUM1* gene specific primers. All *F. verticillioides* strains were positive for the *ITS* region and exhibited the expected 431 bp DNA fragment except *A. flavus*, which was used as control (Figure 1). In addition, all the tested strains of *F. verticillioides* revealed the *FUM1* gene expression and amplified fragment with the molecular size of 183 bp, corresponding to the expected molecular size of the *FUM1* gene sequence, while this band could not be detected in the control sample (*A. flavus*) (Figure 2). Some investigators have used PCR primers from genes directly involved in fumonisin-biosynthesis, like *FUM1*, to identify the fumonisin-producing *Fusarium* species (Abd-Elsalam et al., 2003 ▶; Bluhm et al., 2004 ▶; Patino etal., 2004 ▶). Sreenivasa et al. (2008) ▶ found that, when the isolates of *Fusarium *species were subjected to PCR analysis, the expected 183-bp DNA was amplified in all *F. verticillioides* (45/45), all *F. proliferatum *(04/04), and all *F. anthophilum *(04/04). Amplification was not detected in other isolates of *Fusarium* species (*F. pallidoroseum, F. sporotrichioides,* and* F. oxysporum*)*. *

In order to evaluate the effect of *C. cyminum* oil on expression of *ITS* and *FUM1 *genes encoding proteins involved in fumonisin biosynthesis, *F. verticillioides* isolates were cultured on fumonisin-inducing liquid medium in presence of *C. cyminum* oil (0.195 and 0.390 µl.ml^-1^) at 20°C for 7 days. The fungal mycelia were separated by filtration, then total RNA was extracted and the expression of *ITS* and *FUM1* genes was evaluated by RT-PCR. The results showed that *C. cyminum* oil completely inhibited the expression of *FUM1 *gene in both concentrations dose-dependently (Figure 3).

Based on the statistical analyses, reduction in the expression of fumonisin biosynthetic genes was significant only for *FUM1 *gene (*p*<0.05), while no effect was observed on *ITS* gene. Lopez-Errasquin et al. (2007) ▶ demonstrated a very good linear regression between *FUM1 *transcript levels and fumonisin production using RT-PCR. Despite the known antifungal activity of *C. cyminum*, no information has been reported about its effect on fumonisin biosynthesis in gene expression level. To date, different studies have been conducted to find out bioactive compounds from plants and microorganisms with inhibitory effect on toxigenic fungal growth and mycotoxin production. In a study by Khosravi et al. (2011) ▶, *C. cyminum* essential oil showed significant reductions in values of 94.2% for aflatoxin (AF) B1, 100% for AFB2, 98.9% for AFG1, 100% for AFG2, and 97.5% for total aflatoxin. Another study by Bluma et al. (2008) ▶ showed that thyme and clove essential oils were effective to control aflatoxigenic fungi in stored maize. In conclusion, we reported for the first time that *C. cyminum* oil inhibited expression of* FUM1 *gene in *F. verticillioides* strains. All together, these results indicated that *C. cyminum* may be employed successfully as a good candidate in controlling toxigenic *F. verticillioides* on food and feed and subsequent contamination with fumonisin in practice.

## References

[B1] Abd-Elsalam KA, Aly NI, Abdel-Satar AM, Khalil SM, Verreet AJ (2013). PCR identification of Fusarium genus based on nuclear ribosomal-DNA sequence data. African J Biotech.

[B2] Bluhm BM, Cousin MA, Woloshuk CP (2004). Multiplex real-time PCR detection of fumonisin-producing and trichotheceneproducing groups of Fusarium species. J Food Prot.

[B3] Bluma R, Amaiden MR, Etcheverry M (2008). Screening of argentine plant extracts: Impact on growth parameters and aflatoxin B1 accumulation by Aspergillus section Flavi. Int J Food Microbiol.

[B4] Desjardins AE, Munkvold GP, PlattnerRD, Proctor RH (2002). Fum1: a gene required for fumonisin biosynthesis but not for maize ear rot and ear infection by Gibberella moniliforme in field tests. Mol Plant Microbe Inter.

[B5] (2000). FarmacopeiaBrasileira.

[B6] Gonzalez-Jaen MT, Mirete S, Patino B, Lopez-Errasquın E, Vazquez C (2004). Genetic markers for the analysis of variability and for production of specific diagnostic sequences in fumonisin-producing strains of Fusarium verticillioides. Eur J Plant Pathol.

[B7] Iacobellis NS, Cantore PL, Capasso F, Senatore F (2005). Antibacterial activity of Cuminum cyminum L. and Carum carvi L. essential oils. J Agric Food Chem.

[B8] Jalali-Heravi M, Zekavat B, Sereshti H (2007). Use of gas chromatography–mass spectrometry combined with resolution methods to characterize the essential oil components of Iranian cumin and caraway. J Chromatogr A.

[B9] Janahmadi M, Niazi F, Danyali S, Kamalinejad M (2006). Effects of the fruit essential oil of Cuminum cyminum Linn. (Apiaceae) on pentylene tetrazol induced epileptiform activity in F1 neurones of Helix aspersa. J Ethnopharmacol.

[B10] Jurado M (2006). “Ana´lisis y diagno´stico de especies de Fusarium productoras de toxinas, y supresencia en cerealesespan˜oles.”Ph.D. thesis.

[B11] Jurado M, Marın P, Callejas C, Moretti A, Vazquez C, Gonzalez-Jaen MT (2010). Genetic variability and fumonisin production by Fusarium proliferatum. Food Microbiol.

[B12] Khosravi AR, Shokri H, Minooeianhaghighi M (2011). Inhibition of aflatoxin production and growth of Aspergillus parasiticus by Cuminum cyminum, Ziziphora clinopodioides, and Nigella sativa essential oils. Foodborne Pathog Dis.

[B13] Lopez-Errasquın E, Vazquez C, Jimenez M, Gonza´lez-Jaen MT (2007). Real-time RT-PCR assay to quantify the expression of FUM1 and FUM19 genes from the fumonisin-producing Fusarium verticillioides. J Microbiol Methods.

[B14] Marín P, Magan N, Vázquez C, González-Jaén MT (2010). Differential effect of environmental conditions on growth and regulation of the fumonisin biosynthetic gene FUM1 in the maize pathogens and fumonisin-producers F. verticillioides and F. proliferatum. FEMS Microbiol Ecol.

[B15] Megalla SE, El-Keltawi NEM, Ross SA (1980). A study of antimicrobial action of some essential oil constituents. Herb Pol.

[B16] Mohammadpour M, Moghimipour E, Rasooli I, Fakoor MH, Astaneh AS, Moosaei SS, Jalili Z (2012). Chemical composition and antifungal activity of Cuminum cyminum L. essential oil from Alborzmountain against Aspergillus species. Jundishapur J Nat Pharm Prod.

[B17] Mule G, Susca A, Stea G, Moretti A (2004). Specific detection of the toxigenic species Fusarium proliferatum and F. oxysporum from asparagus plants using primers based on calmodulin gene sequences. FEMS MicrobiolLett.

[B18] Naeini A, Khosravi AR, Chitsaz M, Shokri H, Kamlnejad M (2009). Anti-Candida albicans activity of some Iranian plants used in traditional medicine. J Mycol Med.

[B19] Naeini A, Ziglari T, Shokri H, Khosravi AR (2010). Assessment of growth-inhibiting effect of some plant essential oils on different Fusarium isolates. J Mycol Med.

[B20] National Committee for Clinical Laboratory Standards (2002). Reference Method for Broth Dilution Antifungal Susceptibility Testing of Filamentous Fungi: Approved Standard M38-A.

[B21] Nelson PE, Desjardins AE, Plattner RD (1993). Fumonisins, mycotoxins produced by Fusarium species: Biology, chemistry, and significance. Ann Rev Phytopathol.

[B22] Nostro A, Cellini L, Di Bartolomeo S, Di Campli E, Grande R, Cannatelli MA (2005). Antibacterial effect of plant extracts against Helicobacter pylori. Phytother Res.

[B23] Patino B, Mirete S, Gonzalez-Jaen T, Mule G, Rodriguez TM, Vazquez C (2004). PCR detection assay of fumonisin-producing Fusarium verticillioides strains. J Food Prot.

[B24] Proctor RH, Brown DW, Plattner RD, Desjardins AE (2003). Co-expression of 15 contiguous genes delineates a fumonisin biosynthetic gene cluster in Gibberella moniliformis. Fungal Genet Biol.

[B25] SalehiSurmaghi H (2006). Medicinal plants and phytotherapy.

[B26] Seo J, Proctor RH, Plattner RD (2001). Characterization of four clustered corregulated genes associated with fumonisin biosynthesis in Fusarium verticillioides. Fungal Genet Biol.

[B27] Sreenivasa MY, Dass RS, Charith AP, Janardhana GR (2006). Molecular detection of fumonisin producing Fusarium species of freshly harvested maize kernelsusing polymerase chain reaction (PCR). Taiwania.

[B28] Sreenivasa MY, Dass RS, Raj APC, Janardhana GR (2008). PCR method for the detection of genus Fusarium and fumonisin-producing isolates from freshly harvested sorghum grains grown In Karnataka, India. J Food Safe.

[B29] Zargari A (1994). Medical Plants.

